# Supplementary figures and images for: The maternal hormone in the male brain: Sexually dimorphic distribution of prolactin signalling in the mouse brain
Source: PLoS One. 2018 Dec 20;13(12):e0208960. doi: 10.1371/journal.pone.0208960 (PMC6301622; doi:10.1371/journal.pone.0208960)

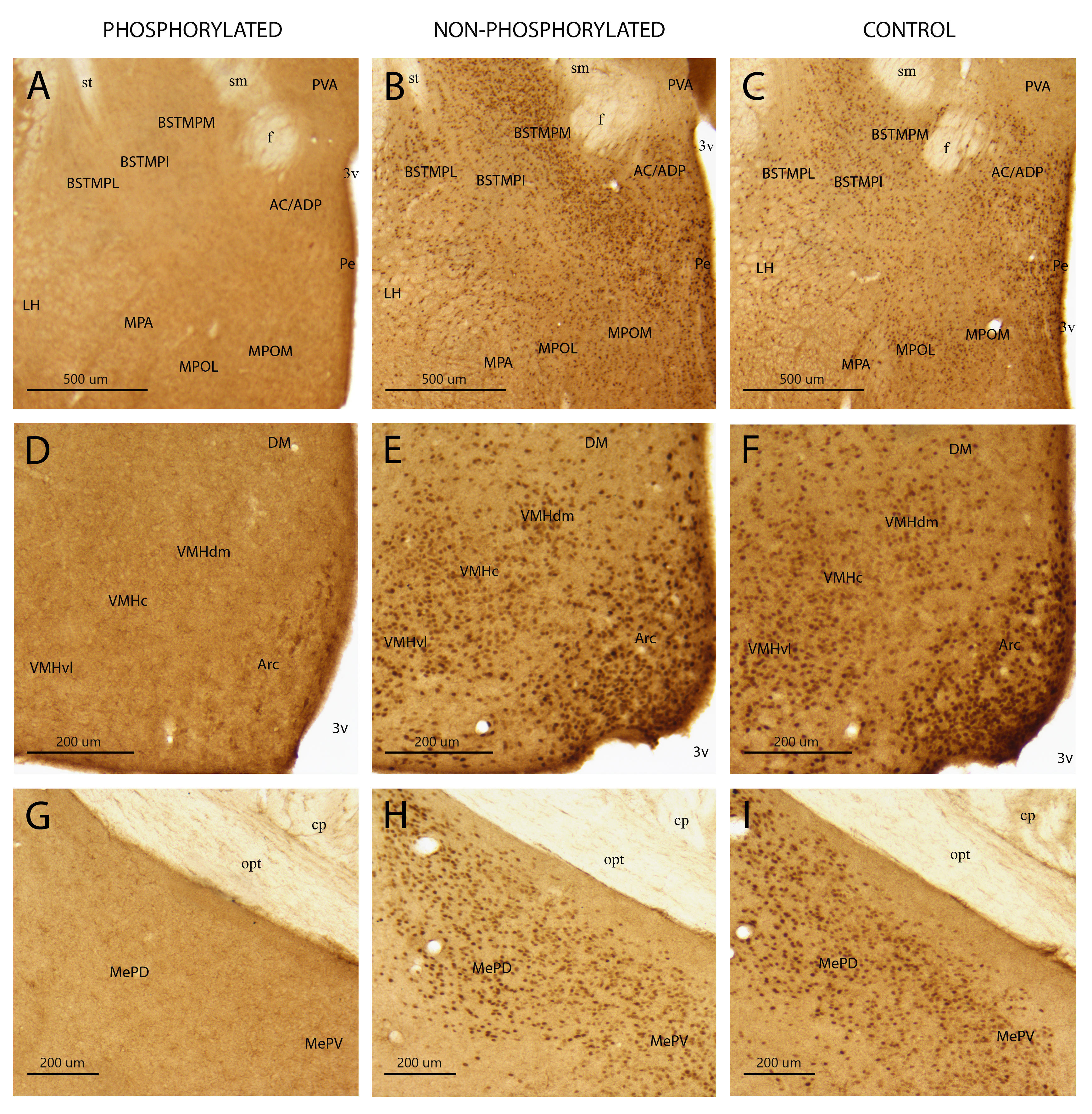

Supplement: S1 Fig — Immunostaining for pSTAT5 was performed using a monoclonal rabbit anti-pSTAT5 primary antibody provided by Cell Signaling Technology (Phospho-Stat5 Tyr694 (D47E7), Catalog number #4322, Danvers, MA). We validated the specificity of this antibody by performing an antibody competition assay in complete series of brain sections from previously studied (pregnant) female mice (Salais-López et al., 2017) known to display high levels of pSTAT5 immunoreactivity. Sections underwent pSTAT5 immunohistochemistry using a primary antibody solution that had been preincubated overnight with the immunogenic peptide against which this antibody was raised (synthetic peptide corresponding to residues surrounding Tyr694 of human STAT5a protein, aminoacid sequence LAKAVDGyVKPQIKQ, where the phospho-tyrosine is indicated in lower case) (left column in the figure). We added this peptide to the primary antibody incubation solution at saturating concentrations (20 times higher than the molar equivalent of the antibody concentration used in the incubation solution). In order to check that this antibody specifically binds the phosphorylated form of STAT5, we included an additional control, consistent in preincubating the antibody with the non-phosphorylated version of the immunogenic peptide (aminoacid sequence LAKAVDGYVKPQIKQ, where the central tyrosine is not phosphorylated) (right column in the Fig). Both peptides were synthesized by the Peptide and Protein Chemistry Lab (Príncipe Felipe Research Center, CSIC, Valencia, Spain). Preincubation with the phosphorylated immunogenic peptide virtually abolished all pSTAT5-ir in the examined tissue (A, D and G, except for some faintly labelled cells in the arcuate nucleus, see D), as compared to sections preincubated with the non-phosphorylated peptide (B, E and H) or to non-preincubated controls (C, F and I). Altogether, this indicates that this primary antibody specifically binds to the phosphorylated form of pSTAT5, thus validating the employed [file pone.0208960.s001.tif]
